# Safety of surveillance endoscopy and EUS of the esophagus after neoadjuvant chemoradiotherapy: Results from the (pre)SANO trial

**DOI:** 10.1055/a-2645-7637

**Published:** 2025-07-31

**Authors:** Sanjiv S.G. Gangaram Panday, Low Kuan Yean, Tanya M. Bisseling, Wouter L. Curvers, Jolanda M. van Dieren, Rutger Quispel, Liekele E. Oostenbrug, Andries van der Linden, Sietske Corporaal, Lieke Hol, Eva Kouw, Jolein van der Kraan, Wouter L. Hazen, Judith Honing, J. Jan B. van Lanschot, Bianca Mostert, Joost J. Nuyttens, Pieter C. van der Sluis, Bas P.L. Wijnhoven, Manon C.W. Spaander, Sjoerd M. Lagarde

**Affiliations:** 1 6993 Surgery, Erasmus MC Cancer Institute, University Medical Center, Rotterdam, Netherlands; 2 6034 Gastroenterology and Hepatology, Radboud University Medical Centre, Nijmegen, Netherlands; 3 3168 Gastroenterology and Hepatology, Catharina Hospital, Eindhoven, Netherlands; 4 1228 Gastrointestinal oncology, The Netherlands Cancer Institute - Antoni van Leeuwenhoek Hospital, Amsterdam, Netherlands; 5 84744 Gastroenterology and Hepatology, Reinier de Graaf Gasthuis, Delft, Netherlands; 6 3802 Gastroenterology and Hepatology, Zuyderland Medical Centre Heerlen, Heerlen, Netherlands; 7 1153 Gastroenterology and Hepatology, ZTG Hospital, Almelo, Netherlands; 8 4480 Gastroenterology and Hepatology, Medical Centre Leeuwarden, Leeuwarden, Netherlands; 9 7000 Gastroenterology and Hepatology, Maasstad Hospital, Rotterdam, Netherlands; 10 240279 Gastroenterology and Hepatology, Gelre Hospitals, Apeldoorn, Netherlands; 11 4501 Gastroenterology and Hepatology, Leiden University Medical Centre, Leiden, Netherlands; 12 7898 Gastroenterology and Hepatology, Elisabeth TweeSteden Hospital, Tilburg, Netherlands; 13 6993 Gastroenterology and Hepatology, Erasmus MC Cancer Institute, University Medical Center, Rotterdam, Netherlands; 14 6993 Medical oncology, Erasmus MC Cancer Institute, University Medical Center, Rotterdam, Netherlands; 15 6993 Radiotherapy, Erasmus MC Cancer Institute, University Medical Center, Rotterdam, Netherlands

**Keywords:** Endoscopy Upper GI Tract, Barrett's and adenocarcinoma, Diagnosis and imaging (inc chromoendoscopy, NBI, iSCAN, FICE, CLE), Endoscopic ultrasonography, Esophageal cancer

## Abstract

**Background and study aims:**

Active surveillance has been proposed for patients with esophageal cancer and a clinical complete response after neoadjuvant chemoradiotherapy (nCRT). This strategy involves repeated esophagogastroduodenoscopy (EGD) with bite-on-bite biopsies and endoscopic ultrasonography (EUS) with fine-needle aspiration (FNA) to detect tumor regrowth or residual disease. The aim of this study was to assess safety of endoscopic procedures during active surveillance.

**Patients and methods:**

A prospective multicenter cohort including patients who were treated with nCRT for esophageal cancer and who underwent EGD with bite-on-bite biopsies and/or EUS (with fine-needle aspiration) was retrospectively analyzed. The primary outcome was the difference in number of serious adverse events (SAEs) in endoscopic procedures performed within vs. more than 3 months after nCRT. The secondary outcome was mechanical injury.

**Results:**

In 920 patients, 2291 endoscopic procedures were performed (57% EGD combined with EUS, 39% EGD only and 4% EUS only). Of these procedures, 819 (36%) were performed more than 3 months after nCRT in 186 patients. Two gastrointestinal bleedings were reported during endoscopic procedures performed within 3 months after nCRT. One gastrointestinal bleeding and two infections were reported after 3 months following nCRT. Frequency of SAEs before and after 3 months following completion of nCRT was not significantly different (2 vs. 3, odds ratio 2.7, 95% confidence interval 0.3–32.4, *P* = 0.36).

**Conclusions:**

EGD with bite-on-bite biopsies and EUS with FNA seem to be safe during an active surveillance strategy in esophageal cancer patients after nCRT.

## Introduction

Following results of the CROSS study, locoregionally advanced esophageal cancer is treated with neoadjuvant chemoradiotherapy (nCRT) followed by surgery 1 . Esophagectomy is a procedure with high morbidity and has persistent major impact on quality of life (QoL) 2 3 . Moreover, in 29% of patients, no viable tumor cells are found in the resection specimen after esophagectomy and a significant number of patients develops early metastases after esophagectomy 4 . Based on this, it was hypothesized that active surveillance is an alternative treatment option, in which patients undergo surgery only if locoregional regrowth is detected in the absence of distant metastases.

The pre Surgery As Needed for Oesophageal cancer (SANO) trial investigated the efficacy of using a combination of diagnostic tests to accurately identify patients who achieved a clinical complete response (CCR) after nCRT 5 . Recent results from the SANO trial showed noninferior 2-year survival for patients undergoing active surveillance compared with patients undergoing standard surgery 6 . Furthermore, short-term global QoL was better in the active surveillance arm 6 . During active surveillance, repeated diagnostic tests are performed to detect residual malignancy with positron emission tomography-computed tomography (PET-CT), esophagogastroduodenoscopy (EGD) with bite-on-bite biopsies, and endoscopic ultrasound (EUS) with fine-needle aspiration (FNA) in case of suspected lymph nodes. EGD and EUS are generally considered safe procedures, with complication rates less than 2.5% in various studies 7 8 9 10 . Previously, the preSANO and SANO trials showed that these endoscopic procedures can be performed without relevant complications within 3 months after nCRT for identification of patients with a CCR 5 11 . However, the safety of these procedures more than 3 months after nCRT is largely uncharted territory and the number of endoscopic evaluations in previous publications on this topic was too small to detect possible complications. It could be hypothesized that the preserved esophagus may become increasingly vulnerable to endoscopic procedures over time due to irradiation-induced chronic ulceration or fibrosis of the mucosal wall leading to stenosis, which could increase risk of perforation 12 .

Therefore, this study aimed to investigate complications associated with EGD and EUS procedures in patients with esophageal cancer after nCRT and to assess differences in safety of endoscopic surveillance in the first 3 months after nCRT versus those performed thereafter in the context of active surveillance.

## Patients and methods

### Study design and patients

A prospective multicenter cohort including patients with esophageal or gastroesophageal junction cancer who underwent nCRT according to CROSS in the preSANO and SANO trials with at least one endoscopic response evaluation was retrospectively analyzed 5 6 . Patients were identified from the trial databases. Endoscopic procedures with regular biopsies were excluded. Because the current study constitutes a substudy of the (pre)SANO trials, which had already received ethical approval from the Medical Ethics Committee of Erasmus MC (MEC-2013–211, MEC-2017–392), no additional ethical approval was necessary.

### Procedures

The first clinical response evaluation was conducted according to a standardized protocol at 4 to 6 weeks after nCRT and consisted of an EGD with at least four bite-on-bite biopsies from the primary tumor location 13 . The bite-on-bite technique involves taking a second biopsy at the same site to sample deeper tissue. When no vital tumor cells were identified, patients were scheduled for a second response evaluation. This consisted of an identical EGD and an EUS with FNA of suspected lymph nodes detected during EUS or on PET-CT at 10 to 12 weeks after nCRT. These endoscopic response evaluations were repeated at 6, 9, 12, 16, 20, 24, 30, 36, 48, and 60 months after completion of nCRT in patients with a CCR who underwent active surveillance in the SANO trial. Surgery was planned when residual tumor was histologically proven or highly suspected and response evaluations were no longer necessary 13 14 . All patients in the preSANO trial and in the surgery arm of the SANO trial were referred for surgery after two response evaluations at 10 to 12 weeks after nCRT.

### Outcomes

The primary outcome was difference in number of serious adverse events (SAEs) in endoscopic procedures performed within 3 months after completion of nCRT versus more than 3 months after nCRT. An SAE was defined as an AE (possibly) related to the intervention (i.e., the endoscopic procedure) requiring hospital admission or extending a hospital admission, and/or that was fatal and/or life-threatening to the patient 13 . SAEs were mandatory for the hospitals to report and were reviewed by the SANO study team. AEs from endoscopic procedures were classified according to the AGREE classification system, in which the severity of AEs is graded with increasing clinical impact from Grade 1 to Grade 5 ( **Supplementary appendix** and **Supplementary Table 1** ) 15 . Grade 1 AEs were not mandatory to report. Secondary outcome was mechanical injury to the mucosa, which was reported if regular biopsies were taken afterwards. These injuries did not require medical intervention. Endoscopic reports and follow-up visits were collected and reviewed by data managers and the SANO study team.

### Statistical analysis

Frequencies and percentages were used to describe baseline clinical and tumor characteristics, incidence of SAEs, and occurrence of mechanical injury. Baseline characteristics were compared using Student’s *t* -test for continuous variables and Chi-squared test for categorical variables. Patients who underwent endoscopic response evaluations after 3 months were also assessed within the 3-month period. Consequently, their data were incorporated into the analysis for both time frames. Fisher’s exact test (expected cell frequencies < 5) was used to identify a statistically significant difference in percentage of SAEs that occurred within 3 months after the end of nCRT versus those that occurred thereafter. Data were analyzed using R version 4.2.2 (R: A Language and Environment for Statistical Computing; The R Foundation for Statistical Computing, Vienna, Austria).

## Results

**Fig. 1:**
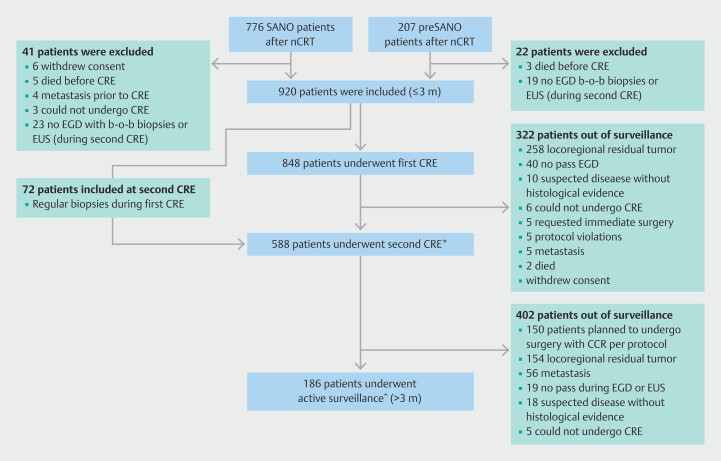
Patient flowcharts for inclusion of patients from the preSANO and SANO trial. b-o-b, bite-on-bite; CCR, clinical complete response; CRE, clinical response evaluation; EGD, esophagogastroduodenoscopy; EUS, endoscopic ultrasound; nCRT, neoadjuvant chemoradiotherapy. *36 patients underwent an extra endoscopic procedure; ^6 patients did not undergo the endoscopic procedure at 6 months after nCRT.

A total of 920 patients underwent at least one clinical response evaluation and were included in the study ( Fig. 1 ). Of these patients, 186 underwent a clinical response evaluation beyond 3 months. Baseline characteristics are shown in Table 1 . Between June 2015 and January 2024, 2291 endoscopic procedures were performed in 14 Dutch hospitals, with a mean (SD) of 4.90 (1.28) bite-on-bite biopsies. These were conducted at a median of 3 months (interquartile range [IQR] 1–9) after completion of nCRT. Among them, there were 1302 EGDs (56%) with bite-on-bite biopsies combined with an EUS during a single session, 896 EGDs (39%) with bite-on-bite biopsies only, and 93 EUSs (4%) only. FNA was performed in 377 of 1395 EUSs (27%) and fine-needle biopsy (FNB) during four EUSs (4/1395, 0.3%). A total of 819 (36%) endoscopic procedures was performed more than 3 months after nCRT ( Table 2 ).

**Table:** **Table 1** Baseline characteristics of study population (≤ or > 3 months after nCRT).

	**Patients** ≤ 3 months N = 920	**≤ 3 months** and > 3 months n = 186	***P* value **
Age, years			0.068
Median	68	69	
IQR	61–73	63–74	
Male sex	760 (83)	146 (79)	0.221
Histology			0.269
Adenocarcinoma	731 (80)	139 (75)	
Squamous cell carcinoma	166 (18)	43 (23)	
Other	23 (3)	4 (2)	
Tumor differentiation grade ^*^			0.530
G1	117 (14)	32 (17)	
G2	392 (47)	90 (48)	
G3	316 (38)	61 (33)	
Could not be determined	15 (2)	3 (2)	
Tumor location			0.615
Proximal esophagus	10 (1)	2 (1)	
Middle esophagus	91 (10)	23 (12)	
Distal esophagus	588 (64)	110 (59)	
Esophagogastric junction	231 (25)	51 (27)	
Clinical T-category			0.960
T1	4 (0)	1 (1)	
T2	178 (19)	35 (19)	
T3	660 (72)	137 (74)	
T4	21 (2)	3 (2)	
Could not be determined	57 (6)	10 (5)	
Clinical N-category			0.298
N0	328 (36)	79 (43)	
N1	333 (36)	66 (36)	
N2	187 (20)	31 (17)	
N3	19 (2)	4 (2)	
Could not be determined	53 (6)	6 (3)	
WHO performance status ^†^			0.519
0	517 (67)	126 (70)	
1	245 (32)	50 (28)	
2	7 (1)	2 (1)	
3	1 (0)	1 (1)	
Percentages may not add up to 100 because of rounding. Characteristics are displayed as number (%), except age (median, IQR). IQR, interquartile range; nCRT, neoadjuvant chemoradiotherapy; WHO, World Health Organization. *80 missing (all preSANO): ≤ 3 months. ^†^ 150 missing (63 preSANO): ≤ 3 months = 143 vs > 3 months = 7.			

**Table:** **Table 2** Endoscopic procedures per time point.

Time point after nCRT (months)	Total endoscopic procedures	SANO	preSANO
1	848*	728	120
3	624	490	134
6	180	180	–
9	122	122	–
12	105	105	–
16	92	92	–
20	76	76	–
24	80	80	–
30	72	72	–
36	64	64	–
48	26	26	–
60	2	2	–
nCRT, neoadjuvant chemoradiotherapy. *72 unique patients were included at the second time point, because regular biopsies were taken at the first time point			

### Primary outcome: Serious adverse events

SAEs were reported for five endoscopic procedures (0.22%). Two patients had gastrointestinal bleeding after EGD with bite-on-bite biopsies, one patient had gastrointestinal bleeding of a preexisting non-malignant gastric ulcer, and two patients had an infection secondary to EUS with FNA/FNB ( Table 3 ). The rate of ≥ Grade 2 gastrointestinal bleeding after EGD caused by bite-on-bite biopsies was 0.09% (2/2198) and the rate of ≥ Grade 2 infection after EUS with FNA/FNB was 0.53% (2/377). No significant difference was found in the number of SAEs between the procedures conducted within 3 months after nCRT and more than 3 months after nCRT (2 vs. 3, odds ratio 2.70 (95% confidence interval 0.31–32.41; *P* = 0.36).

**Table:** **Table 3** SAEs per endoscopic procedure type.

Endoscopic procedure	Total	SAE	Total
EGD with b-o-b biopsies and EUS	2291	Gastrointestinal bleeding due to manipulation	1 (0.04)
EGD with b-o-b biopsies	2198	Gastrointestinal bleeding due to biopsies	2 (0.09)
EUS with FNA	377	Infection due to EUS with FNA/FNB	2 (0.53)
b-o-b, bite-one-bite; EGD, esophagogastroduodenoscopy; EUS, endoscopic ultrasound; FNA, fine-needle aspiration; SAE, serious adverse event.			

### Gastrointestinal bleeding

One patient presented with hematemesis at the Emergency Department (ED) 1 day after EGD with bite-on-bite biopsies at 6 months after nCRT and used apixaban during the EGD. An EGD showed active bleeding at the biopsy site and hemostasis was achieved by use of a endoscopic hemostat. The patient was discharged in good condition after 2 days. The complication was scored as a Grade 3a AE. Another patient used dual antiplatelet therapy (aspirin and ticagrelor) during the EGD and collapsed a few hours after EGD with bite-on-bite biopsies at 1 month after nCRT. The hemoglobin level had dropped from 10 g/dL to 7.1 g/dL, making the patient suspicious for bleeding. The patient was transfused with two units of packed red blood cells (RBCs) and was discharged in good condition after 2 days. The adverse event was defined as a Grade 2. A third patient presented with hematemesis and melena at the emergency department on the day of EGD with bite-on-bite biopsies at 1 month after nCRT. This patient used aspirin during the EGD. An EGD was performed and a bleeding ulcer in the angulus of the stomach (not at the biopsy site) was injected with adrenaline and the vessel was clipped. The patient was transfused with two units of packed RBCs and observed in the Intensive Care Unit for one night. After 4 days, the patient was discharged in good condition. The severity was scored as a Grade 4 AE.

### Infection/inflammation

One patient presented with pain in the abdomen, normal vital parameters, and a C-reactive protein (CRP) level of 100 mg/L at 3 days after EUS with FNA, 8 months after nCRT. This patient was diagnosed with mucositis after EUS-FNA and was admitted to the hospital for 1 day (> 24 hours) without medical intervention, scored as a Grade 2 AE. The second patient presented at the ED 5 days after EUS with FNB at 6 months after nCRT with fever, nausea/vomiting, and diarrhea. The CRP level was 251 mg/L. Vital parameters normalized after administration of intravenous (IV) fluids. Blood cultures were positive for *Streptococcus anginosus* and the patient received IV antibiotics for 5 days. The sepsis was deemed to be related to the EUS with FNB. The patient was discharged in good condition after 4 days and the complication was scored as a Grade 2 AE.

### Secondary outcome: mucosal injury

Four severe mucosal injuries (4/2291, 0.17%) were reported, two of which occurred within 3 months after nCRT and two of which occurred more than 3 months after nCRT. One reported laceration was caused by introduction of the EUS endoscope. The other three injuries were caused by the bite-on-bite biopsy procedures.

## Discussion

In this study, it was demonstrated that endoscopic SAEs are uncommon in patients with esophageal cancer who undergo active surveillance after nCRT. The complication rate recorded in this study was 0.2% for all 2291 endoscopic procedures. Endoscopy-related SAEs during the early (≤ 3 months) and late period (> 3 months) post chemoradiotherapy were similar in the present study (2 vs. 3, *P* = 0.36). Therefore, timing of endoscopic procedures did not seem to affect risk of complications. Three patients developed upper gastrointestinal bleeding after EGD with bite-on-bite biopsies and two patients developed an infection related to EUS with FNA/FNB. There was no reported case of perforation or mortality and all affected patients with complications could be discharged without long-term sequelae.

In previous studies, the safety of endoscopic procedures in the irradiated esophagus was studied no longer than 3 months after nCRT 16 . SAEs did not occur in these endoscopic response evaluations, shortly after nCRT, which supports the hypothesis that EGD and EUS are safe after nCRT. However, safety of endoscopic procedures beyond 3 months after nCRT was not yet established. In watch-and-wait regimes for rectal cancer, patients also undergo repeated endoscopies of the irradiated rectum.

Literature does not report complications of endoscopy in the irradiated rectum 17 18 .

Radiotherapy of the esophagus induces radiation esophagitis, which is exacerbated by the addition of chemotherapy. Radiation esophagitis can be classified as acute (≤ 90 days after completion of radiotherapy) and late (> 90). In the acute phase, radiotherapy acts as a pro-inflammatory signal causing mucosal breakdown and release of pro-inflammatory cytokines, which in turn cause fragility of the mucosal wall 19 . In the later phase, radiotherapy may cause chronic ulceration or fibrosis of the mucosal wall leading to stenosis 12 .

Two of the three patients with upper gastrointestinal bleeding after EGD developed the bleeding within the first 3 months after nCRT. Although our study was not designed to directly compare complication rates between patients with and without neoadjuvant chemoradiotherapy, it is important to contextualize our findings. The observed bleeding rate is in line with current literature, with reported incidences of less than 0.01% after diagnostic endoscopy with biopsies 20 . In two of the three bleeding patients, bleeding was biopsy-related. Biopsy-related bleeding occurred in patients who were on direct oral anticoagulants (DOACs) or dual antiplatelet therapy. DOACs and dual antiplatelet therapy are known risk factors for gastrointestinal bleeding, which is why they were withheld during EUS with FNA, according to the protocol 21 22 . EGD with regular biopsies in the standard population is regarded as a low-risk procedure and can be performed without pausing anticoagulants. Results of the present study suggest that bite-on-bite biopsies in an irradiated esophagus can be regarded as low-risk procedures too. In addition, based on the findings that bite-on-bite biopsies are not necessarily deeper than regular biopsies, the remaining risk may be insignificant 11 . However, use of anticoagulants during EGD with bite-on-bite biopsies was not documented.

Literature reports overall complication rates for regular EUS with FNA of 0.98% and an infection rate of less than 0.05% 23 . In the present study, there were two patients (0.52%) with EUS-FNA/FNB-related complications, both of which were infections. Although comparison of these results with literature is hampered by the low incidence of complications and the relatively small sample size of the present study, risk of AEs does not seem to be increased after nCRT.

An important strength of this study is that data collection was prospective and SAEs were systematically recorded and reported. As compared with previous studies, there were considerably more endoscopic procedures after nCRT including many beyond 3 months, allowing for a more comprehensive assessment of safety during long-term surveillance 5 11 . Furthermore, this was a multicenter study with dedicated endoscopists, simulating real-life practice.

A limitation of the study is that the reporting of (non-serious) AEs was not mandatory. Although all endoscopic reports were reviewed centrally by the SANO study team during follow-up, if an endoscopist did not consider a laceration to be relevant, it might not have been documented. In addition, if SAEs occurred after the procedure and were not noticed by the SANO study team, we primarily had to rely on the hospital to report them. Although it is theoretically possible that some patients were hospitalized for complications at non-affiliated hospitals, this is unlikely to have significantly impacted our data. In the Dutch healthcare system, inter-hospital communication is standard practice, especially for post-procedure complications, making underreporting unlikely. Although these data suggest acceptable complication rates, it is important to realize that safety data on response evaluations beyond 2 years after nCRT remain limited. This might increase with newly implemented active surveillance strategies for esophageal cancer. It can be hypothesized that endoscopic procedures remain safe over a longer term after nCRT in the irradiated esophagus because we do not expect an increase of fibrosis, strictures, or ulceration more than 2 years after nCRT.

## Conclusions

In conclusion, EGD with bite-on-bite biopsies and EUS with FNA are safe procedures to perform during active surveillance strategies within the irradiated esophagus with an acceptable SAE rate. There were no differences in SAE rates for procedures performed within 3 months after nCRT compared with more than 3 months after nCRT.

## Supplementary Material

Supplementary Material Zusatzmaterial

## References

[1] Eyck BM van Lanschot JJB Hulshof M Ten-year outcome of neoadjuvant chemoradiotherapy plus surgery for esophageal cancer: the randomized controlled CROSS Trial J Clin Oncol 2021 39 1995 2004 10.1200/JCO.20.03614 33891478

[2] Noordman BJ Verdam MGE Lagarde SM Impact of neoadjuvant chemoradiotherapy on health-related quality of life in long-term survivors of esophageal or junctional cancer: results from the randomized CROSS trial Ann Oncol 2018 29 445 451 10.1093/annonc/mdx726 29126244

[3] van der Werf LR Busweiler LAD van Sandick JW Reporting national outcomes after esophagectomy and gastrectomy according to the Esophageal Complications Consensus Group (ECCG) Ann Surg 2020 271 1095 1101 10.1097/SLA.0000000000003210 30676381

[4] Shapiro J van Lanschot JJB Hulshof M Neoadjuvant chemoradiotherapy plus surgery versus surgery alone for oesophageal or junctional cancer (CROSS): long-term results of a randomised controlled trial Lancet Oncol 2015 16 1090 1098 10.1016/S1470-2045(15)00040-6 26254683

[5] Noordman BJ Spaander MCW Valkema R Detection of residual disease after neoadjuvant chemoradiotherapy for oesophageal cancer (preSANO): a prospective multicentre, diagnostic cohort study Lancet Oncol 2018 19 965 974 10.1016/S1470-2045(18)30201-8 29861116

[6] van der Wilk BJ Eyck BM Wijnhoven BPL LBA75 Neoadjuvant chemoradiotherapy followed by surgery versus active surveillance for oesophageal cancer (SANO-trial): A phase-III stepped-wedge cluster randomised trial Ann Oncology 2023 34 S1317 41725589

[7] Azam M Hudgi A Uy PP Safety of endoscopy in patients undergoing treatments with antiangiogenic agents: A 5-year retrospective review World J Gastrointest Endosc 2022 14 416 423 36051996 10.4253/wjge.v14.i7.416 PMC9329849

[8] de Moura DTH McCarty TR Jirapinyo P EUS-guided fine-needle biopsy sampling versus FNA in the diagnosis of subepithelial lesions: a large multicenter study Gastrointest Endosc 2020 92 108 119 e103 10.1016/j.gie.2020.02.021 32105712 PMC7340004

[9] Wolfsen HC Hemminger LL Achem SR Complications of endoscopy of the upper gastrointestinal tract: a single-center experience Mayo Clin Proc 2004 79 1264 1267 10.4065/79.10.1264 15473407

[10] Zubarik R Eisen G Mastropietro C Prospective analysis of complications 30 days after outpatient upper endoscopy Am J Gastroenterol 1999 94 1539 1545 10.1111/j.1572-0241.1999.01141.x 10364022

[11] van der Bogt RD van der Wilk BJ Oudijk L Bite-on-bite biopsies for the detection of residual esophageal cancer after neoadjuvant chemoradiotherapy Endoscopy 2022 54 1131 1138 10.1055/a-1846-1025 35668664

[12] Afifi A Powerski M Jechorek D Radiation-induced damage in the upper gastrointestinal tract: clinical presentation, diagnostic tests and treatment options Best Pract Res Clin Gastroenterol 2020 48–49 101711 10.1016/j.bpg.2020.101711 33317797

[13] Noordman BJ Wijnhoven BPL Lagarde SM Neoadjuvant chemoradiotherapy plus surgery versus active surveillance for oesophageal cancer: a stepped-wedge cluster randomised trial BMC Cancer 2018 18 142 10.1186/s12885-018-4034-1 29409469 PMC5801846

[14] Eyck BM van der Wilk BJ Noordman BJ Updated protocol of the SANO trial: a stepped-wedge cluster randomised trial comparing surgery with active surveillance after neoadjuvant chemoradiotherapy for oesophageal cancer Trials 2021 22 345 34001287 10.1186/s13063-021-05274-w PMC8127221

[15] Nass KJ Zwager LW van der Vlugt M Novel classification for adverse events in GI endoscopy: the AGREE classification Gastrointest Endosc 2022 95 1078 1085 e1078 10.1016/j.gie.2021.11.038 34890695

[16] Eyck BM Onstenk BD Noordman BJ Accuracy of detecting residual disease after neoadjuvant chemoradiotherapy for esophageal cancer: A systematic review and meta-analysis Ann Surg 2020 271 245 256 10.1097/SLA.0000000000003397 31188203

[17] Garcia-Aguilar J Patil S Gollub MJ Organ preservation in patients with rectal adenocarcinoma treated with total neoadjuvant therapy J Clin Oncol 2022 40 2546 2556 35483010 10.1200/JCO.22.00032 PMC9362876

[18] van der Valk MJM Hilling DE Bastiaannet E Long-term outcomes of clinical complete responders after neoadjuvant treatment for rectal cancer in the International Watch & Wait Database (IWWD): an international multicentre registry study Lancet 2018 391 2537 2545 10.1016/S0140-6736(18)31078-X 29976470

[19] Adebahr S Schimek-Jasch T Nestle U Oesophagus side effects related to the treatment of oesophageal cancer or radiotherapy of other thoracic malignancies Best Pract Res Clin Gastroenterol 2016 30 565 580 10.1016/j.bpg.2016.07.003 27644905

[20] Sieg A Hachmoeller-Eisenbach U Eisenbach T Prospective evaluation of complications in outpatient GI endoscopy: a survey among German gastroenterologists Gastrointest Endosc 2001 53 620 627 10.1067/mge.2001.114422 11323588

[21] Cheung KS Leung WK Gastrointestinal bleeding in patients on novel oral anticoagulants: Risk, prevention and management World J Gastroenterol 2017 23 1954 1963 10.3748/wjg.v23.i11.1954 28373761 PMC5360636

[22] Gimbel ME Minderhoud SCS Ten Berg JM A practical guide on how to handle patients with bleeding events while on oral antithrombotic treatment Neth Heart J 2018 26 341 351 10.1007/s12471-018-1117-1 29740754 PMC5968004

[23] Wang KX Ben QW Jin ZD Assessment of morbidity and mortality associated with EUS-guided FNA: a systematic review Gastrointest Endosc 2011 73 283 290 21295642 10.1016/j.gie.2010.10.045

