# Supplementary material for: Safety of surveillance endoscopy and EUS of the esophagus after neoadjuvant chemoradiotherapy: Results from the (pre)SANO trial
Source: Endosc Int Open. 2025 Jul 31;13:a26457637. doi: 10.1055/a-2645-7637 (PMC12372426; doi:10.1055/a-2645-7637)
Supplement: Supplementary file 1 — Supplementary Material [file 10-1055-a-2645-7637_26513456.pdf]

Supplementary Table 1 AGREE classification for adverse events in gastrointestinal endoscopy.

Grading	Definition
No adverse event	A telephone contact with the general practitioner, outpatient clinic, or endoscopy service without any intervention or<br>Extended observation of the patient after the procedure, < 3 hours, without any intervention
Grade I	Adverse events with any deviation of the standard postprocedural course, without the need for pharmacologic treatment or endoscopic, radiologic, or surgical interventions<br>Presentation at the emergency ward, without any intervention or<br>Hospital admission (<24 hours), without any intervention or<br>Allowed therapeutic regimens are drugs as antiemetics, antipyretics, analgesics, and electrolytes or<br>Allowed diagnostic tests: radiology and laboratory tests
Grade II	Adverse events requiring pharmacologic treatment with drugs other than those allowed for grade I adverse events (ie, antibiotics, antithrombotics, etc) or<br>Blood or blood product transfusions or<br>Hospital admission for more than 24 hours
Grade III	Adverse events requiring endoscopic, radiologic, or surgical intervention
Grade IIIa	Endoscopic or radiologic intervention
Grade IIIb	Surgical intervention
Grade IV	Adverse events requiring intensive care unit/critical care unit admission
Grade IVa	Single-organ dysfunction (including dialysis)
Grade IVb	Multiorgan dysfunction
Grade V	Death of the patient

Definition of adverse event: all negative outcomes for a patient that prevent completion of the planned procedure or cause any deviation from the standard postprocedural course.
The contents of this table are sourced from Nass et all [15].
Nass KJ, Zwager LW, van der Vlugt M et al. Novel classification for adverse events in GI endoscopy: the AGREE classification. Gastrointest Endosc 2022; 95: 1078-1085 e1078

Endosc Int Open | DOI 10.1055/a-2645-7637 | © The Author(s).

Supplementary Table 2 Members of the SANO study group, other than the manuscript authors.

Name	Department	Center
Arjun D. Koch	Gastroenterology	Erasmus MC – University Medical Centre
Suzan Nikkessen	Gastroenterology	Erasmus MC – University Medical Centre
Ate van der Gaast	Medical oncology	Erasmus MC – University Medical Centre
Roelf Valkema	Nuclear medicine	Erasmus MC – University Medical Centre
Michail Doukas	Pathology	Erasmus MC – University Medical Centre
Lindsey Oudijk	Pathology	Erasmus MC – University Medical Centre
Hester F. Lingsma	Public health	Erasmus MC – University Medical Centre
David van Klaveren	Public health	Erasmus MC – University Medical Centre
Roy S. Dwarkasing	Radiology	Erasmus MC – University Medical Centre
Berend van der Wilk	Surgery	Erasmus MC – University Medical Centre
Ben Eyck	Surgery	Erasmus MC – University Medical Centre
Bo Jan Noordman	Surgery	Erasmus MC – University Medical Centre
Maartje Valkema	Surgery	Erasmus MC – University Medical Centre
Geert J. Bulte	Gastroenterology	Radboud University Medical Centre
Peter D. Siersema	Gastroenterology	Radboud University Medical Centre
Harm Westdorp	Medical oncology	Radboud University Medical Centre
Erik H. Aarntzen	Nuclear medicine	Radboud University Medical Centre
Chella S. van der Post	Pathology	Radboud University Medical Centre
Maartje C. van Rijk	Radiology	Radboud University Medical Centre
Pètra M. Braam	Radiotherapy	Radboud University Medical Centre
Heidi Rütten	Radiotherapy	Radboud University Medical Centre
Marcel Verheij	Radiotherapy	Radboud University Medical Centre
Camiel Rosman	Surgery	Radboud University Medical Centre
Bastiaan Klarenbeek	Surgery	Radboud University Medical Centre
Serge J. Zweers	Gastroenterology	Maasstad Hospital
Lisanne Holster	Gastroenterology	Maasstad Hospital
Ewout F.W. Courrech Staal	Radiology	Maasstad Hospital

Endosc Int Open | DOI 10.1055/a-2645-7637 | © The Author(s).

Karen E. Hamoen	Pathology	Maasstad Hospital
Trudy Rapmund	Surgery	Maasstad Hospital
Erwin van der Harst	Surgery	Maasstad Hospital
Peter-Paul Coene	Surgery	Maasstad Hospital
Huseyin Aktas	Gastroenterology	Ziekenhuisgroep Twente Hospital
Polat Dura	Gastroenterology	Ziekenhuisgroep Twente Hospital
Ronald Hoekstra	Medical oncology	Ziekenhuisgroep Twente Hospital
Ali Agool	Nuclear medicine	Ziekenhuisgroep Twente Hospital
Joop van Baarlen	Pathology	Ziekenhuisgroep Twente Hospital
Ellen M. Hendriksen	Radiotherapy	Ziekenhuisgroep Twente Hospital
Henk Jan Mantel	Surgery	Ziekenhuisgroep Twente Hospital
Marc van Det	Surgery	Ziekenhuisgroep Twente Hospital
Ewout Kouwenhoven	Surgery	Ziekenhuisgroep Twente Hospital
Sana A. Mulder	Gastroenterology	Reinier de Graaf Gasthuis
Arjan J. Verschoor	Medical oncology	Reinier de Graaf Gasthuis
Marc R.J. ten Broek	Nuclear medicine	Reinier de Graaf Gasthuis
René J. Dallinga	Radiology	Reinier de Graaf Gasthuis
Karen J. Neelis	Radiotherapy	Reinier de Graaf Gasthuis
Erlinde de Graaf	Surgery	Reinier de Graaf Gasthuis
Stijn van Esser	Surgery	Reinier de Graaf Gasthuis
Jan Willem Dekker	Surgery	Reinier de Graaf Gasthuis
Thomas R. de Wijkerslooth	Gastroenterology	The Netherlands Cancer Institute
Marieke A. Vollebergh	Medical oncology	The Netherlands Cancer Institute
Emilia C. Owers	Nuclear medicine	The Netherlands Cancer Institute
Annemarieke Bartels-Rutten	Radiology	The Netherlands Cancer Institute
Liudmila L. Kodach	Pathology	The Netherlands Cancer Institute
Francine E.M. Voncken	Radiotherapy	The Netherlands Cancer Institute
Yvonne Hilhorst	Surgery and Gastroenterology	The Netherlands Cancer Institute

Endosc Int Open | DOI 10.1055/a-2645-7637 | © The Author(s).

Marjolein Warmerdam	Surgery and Gastroenterology	The Netherlands Cancer Institute
Johanna van Sandick	Surgery	The Netherlands Cancer Institute
Edward Fiets	Medical oncology	Medical Centre Leeuwarden
Marco B. Polée	Medical oncology	Medical Centre Leeuwarden
Anne Marij G. van Burg	Nuclear medicine	Medical Centre Leeuwarden
Judith Nieken	Pathology	Medical Centre Leeuwarden
Rinze Wolf	Radiology	Medical Centre Leeuwarden
Vera Oppedijk	Radiotherapy	Medical Centre Leeuwarden
Marloes Emous	Surgery	Medical Centre Leeuwarden
Daniel A. Hess	Surgery	Medical Centre Leeuwarden
Jean Pierre Pierie	Surgery	Medical Centre Leeuwarden
Willemien Erkelens	Gastroenterology	Gelre Hospital
S. Cathrien S. Tromp – van Driel	Medical Oncology	Gelre Hospital
Marc D. Zuijdwijk	Nuclear Medicine	Gelre Hospital
H. Doornewaard	Pathology	Gelre Hospital
Karin Muller	Radiotherapy	Gelre Hospital, Radiotherapiegroep Deventer
Peter van Duijvendijk	Surgery	Gelre Hospital
Eelco B. Wassenaar	Surgery	Gelre Hospital
Edwin van der Zaag	Surgery	Gelre Hospital
Geert-Jan Creemers	Medical Oncology	Catharina Hospital
Mark J. Roef	Nuclear Medicine	Catharina Hospital
Ineke van Lijnschoten	Pathology	Catharina Hospital
Joost Nederend	Radiology	Catharina Hospital
Maurice J.C. van der Sangen	Radiotherapy	Catharina Hospital
Tom C.G. Budiharto	Radiotherapy	Catharina Hospital
Fanny F.B.M. Heesakkers	Surgery	Catharina Hospital

Endosc Int Open | DOI 10.1055/a-2645-7637 | © The Author(s).

Misha Luyer	Surgery	Catharina Hospital
Grard Niewenhuijzen	Surgery	Catharina Hospital
Fabienne A.R.M. Warmerdam	Medical oncology	Zuyderland Medical Centre
Wendy Schreurs	Nuclear Medicine	Zuyderland Medical Centre
Bart de Vries	Pathology	Zuyderland Medical Centre
Roy F.A. Vliegen	Radiology	Zuyderland Medical Centre
Jeroen Buijsen	Radiotherapy	Zuyderland Medical Centre
Ilse Stohr	Surgery	Zuyderland Medical Centre
Eric H.J. Belgers	Surgery	Zuyderland Medical Centre
Meindert Sosef	Surgery	Zuyderland Medical Centre
Marije Slingerland	Medical Oncology	Leiden University Medical Centre
Richard Raghoo	Nuclear Medicine	Leiden University Medical Centre
A. Stijn L.P. Crobach	Pathology	Leiden University Medical Centre
Aart J. van der Molen	Radiology	Leiden University Medical Centre
Susan J.C.L.M. Quix	Surgery	Leiden University Medical Centre
Wobbe O. de Steur	Surgery	Leiden University Medical Centre
Henk Hartgrink	Surgery	Leiden University Medical Centre
Laurens V. Beerepoot	Medical Oncology	Elisabeth Tweesteden Hospital
David E. Ploeg	Pathology	Elisabeth Tweesteden Hospital
Tom Rozema	Radiotherapy	Elisabeth Tweesteden Hospital
Ilse A.C. Vermeltfoort	Nuclear Medicine	Elisabeth Tweesteden Hospital
Walther Jansen	Surgery	Elisabeth Tweesteden Hospital
Joos Heisterkamp	Surgery	Elisabeth Tweesteden Hospital

Endosc Int Open | DOI 10.1055/a-2645-7637 | © The Author(s).
